# Carbon dots induce pathological damage to the intestine via causing intestinal flora dysbiosis and intestinal inflammation

**DOI:** 10.1186/s12951-023-01931-1

**Published:** 2023-05-25

**Authors:** Mengmeng Jia, Bingcheng Yi, Xian Chen, Yongzhi Xu, Xinkai Xu, Zhaoxu Wu, Jing Ji, Jinglong Tang, Dianke Yu, Yuxin Zheng, Qihui Zhou, Yanjie Zhao

**Affiliations:** 1 School of Public Health, Qingdao University, Qingdao, 266071 China; 2 School of Rehabilitation Sciences and Engineering, University of Health and Rehabilitation Sciences, Qingdao, 266071 China; 3 School of Stomatology, Qingdao University, Qingdao, 266003 China; 4 Zhejiang Engineering Research Center for Tissue Repair Materials, Wenzhou Institute, University of Chinese Academy of Sciences, Wenzhou, 325000 Zhejiang China

**Keywords:** Carbon dots, Probiotics, Reactive oxygen species, Intestinal flora, Inflammation

## Abstract

**Background:**

Carbon dots (CDs), as excellent antibacterial nanomaterials, have gained great attention in treating infection-induced diseases such as periodontitis and stomatitis. Given the eventual exposure of CDs to the intestine, elucidating the effect of CDs on intestinal health is required for the safety evaluation of CDs.

**Results:**

Herein, CDs extracted from ε-poly-l-lysine (PL) were chosen to explore the modulation effect of CDs on probiotic behavior in vitro and intestinal remodeling in vivo. Results verify that PL-CDs negatively regulate *Lactobacillus rhamnosus* (*L. rhamnosus*) growth via increasing reactive oxygen species (ROS) production and reducing the antioxidant activity, which subsequently destroys membrane permeability and integrity. PL-CDs are also inclined to inhibit cell viability and accelerate cell apoptosis. In vivo, the gavage of PL-CDs is verified to induce inflammatory infiltration and barrier damage in mice. Moreover, PL-CDs are found to increase the Firmicutes to Bacteroidota (F/B) ratio and the relative abundance of *Lachnospiraceae* while decreasing that of *Muribaculaceae*.

**Conclusion:**

Overall, these evidences indicate that PL-CDs may inevitably result in intestinal flora dysbiosis via inhibiting probiotic growth and simultaneously activating intestinal inflammation, thus causing pathological damage to the intestine, which provides an effective and insightful reference for the potential risk of CDs from the perspective of intestinal remodeling.

**Supplementary Information:**

The online version contains supplementary material available at 10.1186/s12951-023-01931-1.

## Introduction

Carbon dots (CDs), as newly emerged carbon-based nanomaterials, have been extensively used in bioimaging, drug delivery, and antibacterial applications owing to their excellent optical properties, high stability, and small particle size [1–4]. Especially in curing the infected tissues, lysine-based CDs were confirmed to perform antibacterial effects against *Escherichia coli* (*E. coli*) and *Staphylococcus aureus* (*S. aureus*) through the positive charge of amino acids and the excited reactive oxygen species (ROS), which effectively alleviates the bacterial infection and promotes skin wound healing [5]. Fucoidan-derived CDs were demonstrated to be capable of inhibiting the biofilm formation of *Enterococcus faecalis* by stimulating ROS production, thus effectively treating persistent endodontic infections [6]. Attributing to the excellent therapeutic effect on anti-infection, the extensive use of CDs in medical applications (e.g., persistent endodontic infections, periodontitis, and stomach ulcer [7–9]) is speculated to result in the inevitable exposure of most CDs to the intestine that contains abundant intestinal flora. Additionally, the heat-processed foods (e.g. barbecue, bakery) we regularly eat in our daily diet similarly include a lot of CDs [10, 11]. However, whether and how they affect the balance of intestinal flora and intestinal remodeling remains unknown. Moreover, the stability of CDs was essentially not significantly different when exposed to different pH environments [12], so it is reasonable to speculate that CDs have excellent stability in the stomach or intestine. Therefore, fully elucidating the effect of CDs on intestinal health is urgently required.

Intestinal flora dynamically regulates intestinal homeostasis and maintains intestinal health via balancing the interaction between harmful bacteria and probiotics [13–15]. Dysbiosis of the intestinal flora is accompanied by the altered metabolic functions involved in carbohydrate, lipid, and amino acids, which contributes to intestinal damage [16]. During the intestinal remodeling, probiotics are verified to exert beneficial effects on maintaining intestinal fitness [17], because they not only produce the short-chain fatty acids (SCFAs) to acidify the intestinal environment and inhibit the survival of pathogens [18], but also colonize in the intestinal mucosa to facilitate the nutritional exchange in the intestine flora [19]. For example, probiotics were confirmed to adhere to intestinal epithelial cells and stimulate host immune responses to restore the intestinal barrier [20, 21]. They also produce organic acids (acetic acid, lactic acid, etc.) to inhibit the growth of gram-negative pathogenic bacteria, which plays a preventive and therapeutic role in inflammatory bowel disease (IBD) [22, 23]. Hence, considering the unique antibacterial property of CDs which can inhibit harmful bacteria growth, clarifying the effect and mechanism of CDs on intestinal flora from the perspective of probiotics is necessary.ε-Poly-l-lysine (PL), a natural cationic peptide, exhibits some critical merits in biomedical applications, including anti-bacteria, heat resistance and biodegradability [24, 25]. Interestingly, PL-based CDs (i.e., PL-CDs) remain an excellent anti-bacterial ability due to the retention of large amounts of -NH_2_, and nowadays have been extensively applied in the fields of antibacterial, anti-angiogenic and bacterial imaging [26]. Therefore, PL-CDs were chosen as the representative CDs applied in the biomedical area, and the regulatory effect of PL-CDs on probiotic growth and intestinal remodeling was well investigated in this study. Firstly, PL-CDs were fabricated and the related physicochemical properties were evaluated. Then, the influence of PL-CDs on probiotic growth and the underlying mechanisms were assessed by analyzing the growth, morphology, secreta, and membrane permeability of probiotics. After observation of the cytotoxicity of PL-CDs, the regulatory role of PL-CDs in intestinal remodeling was analyzed in vivo through pathological analysis, molecular biology detection and 16S rRNA sequencing.

## Materials and methods

### Preparation and characterization of PL-CDs

PL powders (Molecular weight: 4000, Shanghai Dipper Biotechnology Co. Ltd, China) were cauterized in a crucible at 240 °C for 3 h. After natural cooling at room temperature, the residue was dissolved in 20 mL of deionized water, and then sonicated for 40 min, followed by centrifugation at 11,000 rpm for 30 min. After that, the supernatant was filtered through a 0.22 μm filter and dialyzed for 24 h in dialysis membranes (molecular weight cut-off: 5000 Da, Shanghai Yuanye Biotechnology Co., Ltd, China). Lastly, the obtained solution was lyophilized to get PL-CDs.

To analyze the size and shape of samples, PL-CDs were dispersed in deionized water and sonicated for 30 min, and then the morphology of PL-CDs was tested by transmission electron microscope (TEM, Mic JEM-1200EX, Japan). Based on the obtained TEM images, the particle diameter (n = 100) was analyzed using ImageJ software. Furthermore, zeta potentials of PL and PL-CDs were measured with Zetasizer Nano ZSE (Malvern Instruments Ltd., UK). Ultraviolet–visible (UV–vis) spectra of PL-CDs was examined by UV–vis Spectrophotometer (SHIMADZU, Japan). The functional groups of PL and PL-CDs were detected using Fourier transform infrared (FT-IR) spectrometer (Thermo Fisher Scientific, USA) over the wavenumber range of 4000–400 cm^−1^, in which data collection was performed by the accumulation of 32 scans with a resolution of 4 cm^−1^.

### Antibacterial assays of PL-CDs against probiotics

#### Probiotic culture

*Lactobacillus rhamnosus* (*L. rhamnosus*, China Center for Type Culture Collection), as a probiotic strain, was cultured in Man-Rogosa-Sharpe (MRS, Solarbio Science & Technology Co. Ltd, China) medium at 37 °C. To activate the frozen *L. rhamnosus*, the inoculation loop was cauterized, cooled, and dipped into the bacterial solution. Then the dipped probiotics were scribed on MRS agar medium in a gradient.

#### Probiotic growth affected by PL-CDs

The effect of PL-CDs on the growth of *L. rhamnosus* was examined by the flat colony counting method [27]. Firstly, *L. rhamnosus* was suspended in MRS at 37 °C overnight and the density was adjusted to 10^7^ Colony-Forming Units per milliliter (CFU/mL). Then, *L. rhamnosus* was incubated with PL-CDs at different concentrations (0, 50, 100, 200, and 400 μg/mL) for 24 and 48 h. After that, the probiotic suspension was diluted 10^6^ times in gradient, and 20 µL was taken out to evenly coat on MRS agar medium. After the incubation, the colonies were recorded and counted. To observe the real-time growth of probiotics, PL-CDs solutions with different concentrations (0, 50, 100, 200, 400 μg/mL) were obtained by dissolving PL-CDs in MRS medium and then were incubated with *L. rhamnosus* (10^7^ CFU/mL) for 24 h at 37 °C. The growth curve of *L. rhamnosus* was obtained by measuring the absorbance at 600 nm with a microplate reader (Thermo Fisher Scientific, China) every 2 h.

PH variation of the probiotics-cultured medium was also analyzed. Briefly, *L. rhamnosus* (10^7^ CFU/mL) was incubated with different PL-CDs solutions (0, 50, 100, 200, 400 μg/mL) for 24 h at 37 °C. Then, the pH value of the probiotic suspension was detected per 6 h by a pH meter (Sartorius, Germany).

Given the photodynamic properties of CDs to enhance the antibacterial property [28], the effect of PL-CDs on the growth of *L. rhamnosus* was observed under light and dark conditions. Briefly, *L. rhamnosus* (10^7^ CFU/mL) was incubated with PL-CDs at different concentrations (0, 50, 100, 200, 400 μg/mL) under light or dark conditions at 37 °C for 24 h. Then the probiotic suspension was diluted 10^6^ times in gradient and 20 μL was uniformly covered on MRS agar medium, and the colony number was recorded and calculated.

#### Probiotic morphology affected by PL-CDs

The effect of PL-CDs on the probiotic morphology was observed by scanning electron microscope (SEM, VEGA3, TESCAN, Czech). Briefly, PL-CDs were incubated with probiotics (10^7^ CFU/mL) for 24 h at 37 °C. After that, the solutions were centrifuged at 4000 rpm for 10 min and then washed twice with PBS. Next, probiotic precipitation was fixed with 2.5% glutaraldehyde at 4 °C overnight. After being dehydrated with different concentrations of alcohol and dried at the critical point of liquid CO_2_. Samples were sprayed with gold to increase conductivity and then observed under SEM. Based on the SEM images, the elongation (the ratio of length and width) of probiotics (n = 50) was measured by ImageJ software.

The probiotic structure of *L. rhamnosus* was also analyzed by TEM (Mic JEM-1200EX, Japan). Briefly, PL-CDs were incubated with probiotics (10^7^ CFU/mL) for 24 h at 37 °C. After that, the solutions were centrifugated at 3500 rpm for 5 min and the precipitates were collected. After being washed twice with PBS, the precipitates were fixed using 2.5% glutaraldehyde overnight at 4 °C. Next, the fixed probiotics were collected by centrifugation and washed 3 times with PBS. Then, the probiotics were fixed in 1% osmium tetroxide solution for 1 h and dehydrated gradually by 30%, 50%, 70%, 90%, and 100% ethanol for 10 min. After that, the probiotics were embedded in epoxy resin and stained with uranyl acetate and lead citrate solution. Lastly, samples were placed on a copper grid, and then the probiotic structure was observed by TEM.

#### Reactive oxygen species (ROS) production detected by DPBF and DCFH-DA probes

1,3-Diphenylisobenzofuran (DPBF, Sinopharm Chemical Reagent Co., Ltd, China) was used as a singlet oxygen (^1^O_2_) probe to measure ROS production. DPBF powder was dissolved in acetonitrile to obtain 2 mg/mL solution. PL-CDs (400 μg/mL) were dissolved in anhydrous ethanol and sonicated. Then PL-CDs and DPBF were mixed in a 99:1 volume. The solution was divided equally into two parts, which were completely away from light or exposed to visible light for 30 min, respectively. Then the spectra were detected every 5 min at 350–600 nm using a microplate reader (BioTek, USA).

ROS levels in probiotics were measured using a 2′,7′-Dichlorodihydrofluorescein diacetate (DCFH-DA) probe (Solarbio Science & Technology Co., Ltd, China). Briefly, *L. rhamnosus* was incubated with different PL-CDs solutions (0, 50, 100, 200, and 400 μg/mL) for 24 h. After the centrifugation of 1 mL suspension, the precipitates were obtained and then incubated with 1 mL DCFH-DA diluted solution (1:1000) for 20 min. Then, the fluorescence intensity of samples under excitation wavelength at 488 nm and emission wavelength at 525 nm was evaluated with the microplate reader.

#### Antioxidant enzyme activity affected by PL-CDs

Catalase (CAT) activity of probiotics was measured by CAT Activity Assay Kit (Solarbio, China) according to the instructions. In brief, *L. rhamnosus* (10^7^ CFU/mL) was incubated with PL-CDs at different concentrations (0, 50, 100, 200, and 400 μg/mL) for 24 h, followed by centrifugation to obtain the precipitates. The CAT extraction solution was added to obtain a concentration of *L. rhamnosus* at 5 × 10^6^ cells/mL and then crushed by an ultrasonic homogenizer (JY92-IIDN, NingBo Scientz Biotechnology Co. Ltd., China) at the power of 200 W for 3 s (10 s of interval time, 30 times of repetition). After being centrifuged at 8000×*g* for 10 min, the supernatant was collected and mixed with CAT working solution to measure the absorbance at 240 nm using the microplate reader.

Glutathione peroxidase (GSH-PX) activity of probiotics was also performed by GSH-PX Activity Assay Kit (Nanjing Jiancheng Bioengineering Institute, China) according to the instructions. Similarly, *L. rhamnosus* (10^7^ CFU/mL) was incubated with PL-CDs at different concentrations (0, 50, 100, 200, and 400 μg/mL) for 24 h. After the centrifugation, the precipitates were sonicated at the power of 200 W for 3 s (10 s of interval time, 30 times of repetition) and recentrifuged at 3000 rpm for 10 min. Then, the supernatant was mixed with the GSH-PX working solution and the absorbance at 412 nm was measured by the microplate reader.

#### Evaluation of lipid peroxidation in probiotic membranes

Malondialdehyde (MDA) test kit (Nanjing Jiancheng Bioengineering Institute, China) was used to evaluate the effect of PL-CDs on lipid peroxidation in probiotic membranes. Briefly, *L. rhamnosus* (10^7^ CFU/mL) was incubated with PL-CDs at different concentrations (0, 50, 100, 200, and 400 μg/mL) for 24 h. After the centrifugation and sonication, the supernatant was mixed with the MDA working solution according to the manufacturer's instructions. Then, the mixed solution was placed in a water bath at 95 °C for 40 min. After centrifugation at 4000 rpm for 10 min, the supernatant was taken and the OD_532_ was measured by the microplate reader.

#### Membrane permeability of probiotics affected by PL-CDs

The effect of PL-CDs on probiotic membrane permeability was evaluated by o-nitrophenyl β-galactoside (ONPG, Aladdin Industrial, China). PL-CDs at different concentrations (0, 50, 100, 200, and 400 μg/mL) were incubated with *L. rhamnosus* (10^7^ CFU/mL) for 24 h. Then, 15 μL of the probiotic suspension was mixed with 110 μL PBS, 15 μL ONPG solution (12.5 mM), and 10 μL DMSO, followed by the incubation of 30 min at 37 °C. Lastly, 20 μL of Na_2_CO_3_ solution (1 M) was added to stop the reaction, and OD_420_ was detected with the microplate reader.

To analyze the DNA content of probiotics affected by PL-CDs, PL-CDs at different concentrations (0, 50, 100, 200, 400 μg/mL) were incubated with *L. rhamnosus* (10^7^ CFU/mL) for 24 h. Then, the DNA released from damaged probiotics was extracted with Bacterial Genome DNA Extraction Kit (Meilunbio, Dalian, China), and the supernatant obtained by centrifugation was used to detect OD_260_ with NanoDrop (Thermo Fisher Scientific, Beijing, China). To visually observe the DNA content, 0.7% agarose gel electrophoresis was performed. Briefly, 0.7% agarose gel was obtained by dissolving the agarose powder (BioFroxx, Germany) into 50 mL 1× TAE buffer (Meilunbio, China) at high temperature. After the temperature of the gel dropped to approximately 65 °C, 5 μL of nucleic acid electrophoresis dye (Meilunbio, Dalian, China) was introduced into the gel and then the mixed solution was poured into the electrophoresis tank. After 30 min, the extracted DNA solution was electrophoresed at a constant voltage of 110 V. Lastly, images were acquired with an Automatic Gel Imaging Analysis System (Peiqing Science and Technology Co., Ltd, Shanghai).

### Cytotoxicity of PL-CDs

#### Cell culture

Caco-2 cells were provided by Prof. Chen Wen of Sun Yat-sen University, and L929 cells were acquired from the Institute for Translation Medicine of Qingdao University, respectively. All cells were free of mycoplasma infection and cultured in DMEM medium (BasalMedia, China) containing 10% fetal bovine serum (Biological Industries, Israel) and 1% penicillin/streptomycin (BasalMedia, China) in a 37 °C incubator with 5% CO_2_.

#### Cell viability

Cell viability of Caco-2 and L929 cells was assessed by Calcein-AM/PI Staining Kit (Meilunbio, China). In brief, cells were seeded in 24-well plates at a density of 2 × 10^4^ cells/well. After 24 h, PL-CDs were added to incubate cells for another 24 h. Then, 300 μL of staining working solution containing Calcein-AM (2 μM) and PI (8 μM) was added and incubated at 37 °C for 30 min in dark conditions. Lastly, the fluorescence images were observed using the Olympus inverted fluorescence microscope (Olympus, Japan). The proportion of living cells (i.e., the ratio of living cells to total cells) was counted by ImageJ.

#### Cell apoptosis

To analyze the cell apoptosis of Caco-2 and L929 cells affected by PL-CDs, 2 × 10^4^ cells were seeded into 24-well plates for 24 h, and then PL-CDs at different concentrations (0, 50, 100, 200, 400 μg/mL) were incubated with the cells for 24 h. After being washed with PBS solution, the cells were collected and diluted to 1 × 10^6^ cells/mL by 1× Binding buffer working solution. Next, 100 μL of the cell suspension was used to perform annexin V-FITC/PI Apoptosis Detection Kit (Meilunbio, Dalian, China) according to the instructions. Lastly, cell apoptosis was analyzed by Flowcytometry (Beckman Coulter, USA).

### Animal treatment

5-week-old male C57BL/6 mice were purchased from Vital River Laboratory Animal Technology Co., Ltd (Beijing, China). The mice were reared at a temperature of 20–26 °C and relative humidity of 40–70%. All mice were randomly divided into three groups (i.e., Control, 10 mg/kg, and 50 mg/kg groups) with four mice in each group. After 5 days of acclimatization, mice were gavaged with saline, 10 or 50 mg/kg PL-CDs every 2 days, and the weight of mice was real-time recorded. On the 16th day, the mice were sacrificed, and the colon, heart, liver, spleen, lung, kidney, brain, and thymus were weighed to calculate the organ coefficients (organ weight/mice weight * 100), respectively. Meanwhile, the cecum contents and a portion of the colon were collected under aseptic conditions. Then, the tissues were placed quickly in liquid nitrogen and subsequently transferred to − 80 °C for subsequent analysis, while the other portion of the colon was fixed in 4% paraformaldehyde for pathological detection. All animal experimental protocols were performed following the Qingdao University Guide for the Care and Use of Laboratory Animals and were approved by the Ethics Committee Medical College of Qingdao University (No. QDU-AEC-2022370).

#### Pathological analysis of colon

For pathological detection, tissues fixed in 4% paraformaldehyde were embedded in paraffin and cut into 6 μm ultrathin sections using a microtome (Leica 2255, Leica Biosystems, Buffalo Grove, USA). Then sections were placed in a 37 °C oven overnight and stained with hematoxylin and eosin (H&E, Beyotime, China) according to product instructions. To visualize the glycoproteins and goblet cells, Periodic acid-Schiff (PAS, Solarbio, China) and Alcian blue-periodic acid-Shiff (AB-PAS, Servicebio, China) stainings were also performed on colon sections according to the manufacturer’s instructions.

#### Quantitative real-time polymerase chain reaction (qRT-PCR)

qRT-PCR was performed according to the previously described protocol [29]. In brief, total RNA from colon tissue was extracted with Invitrogen TRIzol Reagent (Invitrogen, USA). Then, the obtained RNA was reverse-transcribed into cDNA templates using the PrimeScript RT kit (TaKaRa, Japan). TB Green premix Ex *Taq*II (TaKaRa, Japan) was used for qRT-PCR detection via the LightCycler 480 system (Roche, Basel, Switzerland). The primer sequences were shown in Additional file 2: Table S1. Relative mRNA levels were normalized to GAPDH by 2^−ΔΔCt^ method.

#### Western blot

20 mg colon tissue was lysed with 200 μL radioimmunoprecipitation (RIPA) lysis solution. The subsequent protocols were performed as previously described [30]. Briefly, total protein was separated by sodium dodecyl sulfate-polyacrylamide gel electrophoresis (SDS-PAGE) and transferred to polyvinylidene fluoride (PVDF) membranes (Millipore, MA). Membranes were blocked with 5% skim milk and incubated with primary antibodies against zonula occludens-1 (ZO-1, Abcam, USA), occludin (Abcam, USA), and β-actin (Affinity, USA) overnight. Then, horseradish peroxidase (HRP)-conjugated secondary antibodies (Boster, China) were used to incubate the membranes for 2 h. After that, the membranes were visualized with an ECL kit (Boster, China).

#### Enzyme-linked immunosorbent assay (ELISA)

To analyze the inflammatory responses, the colons were ground and centrifuged to obtain the supernatant for subsequent assays. Then, interleukin (IL)-1β, tumor necrosis factor (TNF)-α, IL-10, and interferon (IFN)-γ were measured by ELISA kits (Boster, China) according to the manufacturer's protocol. The standard curve was performed by CurveExpert software.

#### 16S rRNA sequencing

To analyze the regulation of PL-CDs in the intestinal flora, the collected cecum contents were stored in dry ice and 16S rRNA sequencing was performed by Wuhan Service Biotechnology Co. Ltd. Briefly, the primers of 16S rRNA V3-V4 region were designed for PCR amplification with the forward sequence: 5′-ACTCCTACGGGAGGCAGCA-3′ and the reverse sequence: 5′-GGACTACHVGGGTWTCTAAT-3′. Then, the Illumina Novaseq sequencing platform was performed to double-end the sequence of DNA fragments, and trimmomatic v 0.33 and cutadapt 1.9.1 software were used for the quality filtering of the samples. Usearch v10 software was applied for double-ended sequence splicing. The dada2 method in Quantitative Insights into Microbial Ecology 2 (QIIME2) (2020.6) software was performed to denoise and remove the chimeric sequences to obtain high-quality sequences. Operational taxonomic units (OTUs) of high-quality sequences with 97% similarity were analyzed for alpha diversity. Based on the OTUs clustering, species annotation was applied to representative sequences of OTUs to obtain the corresponding species information and abundance distribution.

### Statistical analysis

All data are presented as mean ± standard deviation (SD). Student’s *t-test* was used to determine the difference between the two groups. Differences between multiple groups were conducted using one-way ANOVA. Asterisks represent significant differences with *p < 0.05, **p < 0.01, and ***p < 0.001.

## Results and discussion

### Successful preparation of PL-CDs

**Fig. 1:**
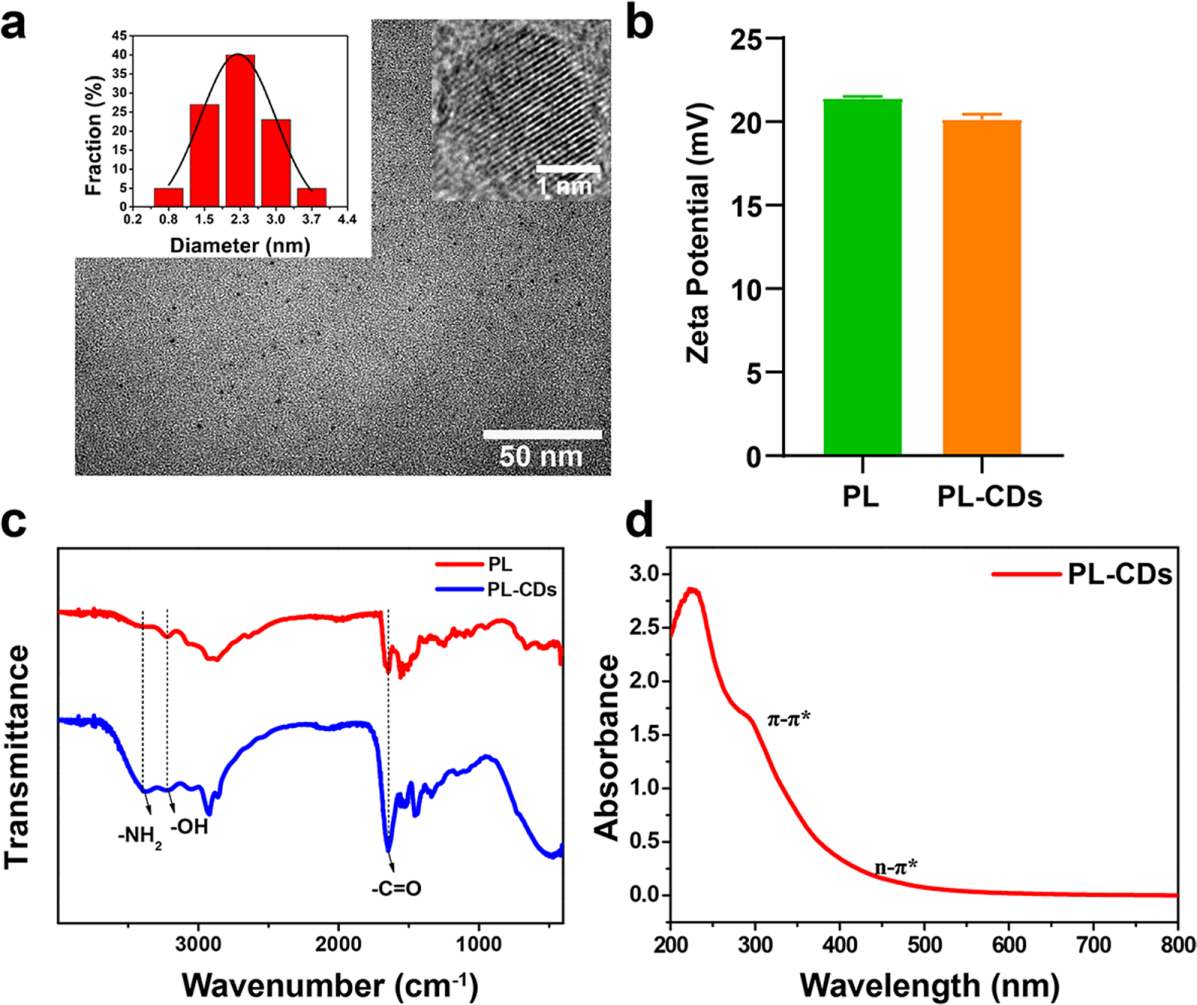
Successful preparation and characterization of PL-CDs. **a** TEM image of PL-CDs. Insets are the particle size distribution and high-resolution TEM (HRTEM) image of PL-CDs. **b** Zeta potential of PL and PL-CDs. **c** FT-IR spectra of PL and PL-CDs. **d** UV–vis spectra of PL-CDs

PL-CDs, prepared by pyrolysis according to the previous report [31], exhibited a sphere-like shape with an average size of 2.21 ± 0.64 nm and uniform dispersion (Fig. 1a). Compared with the positive charge (21.4 ± 0.1 mV) of PL, no significant difference in the zeta potential was found in PL-CDs (20.1 ± 0.3 mV) (Fig. 1b), attributed to the preservation of abundant -NH_2_ groups [32]. In the FTIR spectra of PL-CDs, the observed peak at 1653 cm^−1^ belonged to the C=O bond [33], and the absorption peak at 3600–3000 cm^−1^ was related to the stretching vibration of O–H [34]. The appearance of absorption peaks at 3390 cm^−1^ indicated the successful preparation of PL-CDs (Fig. 1c), which maintain the plentiful –NH_2_ groups for anti-bacteria after the pyrolysis [31]. To confirm the successful fabrication of PL-CDs, the UV–vis spectra of PL-CDs was analyzed and results showed that π–π* leap in the aromatic sp^2^ structural domain (C=C and C–C) resulted in a distinct absorption peak at 270 nm and the n–π* leap in C=O led to a shoulder peak at 300–400 nm (Fig. 1d), reconfirming the main characteristics of CDs [35].

### PL-CDs inhibit probiotic growth in a concentration-dependent manner

**Fig. 2:**
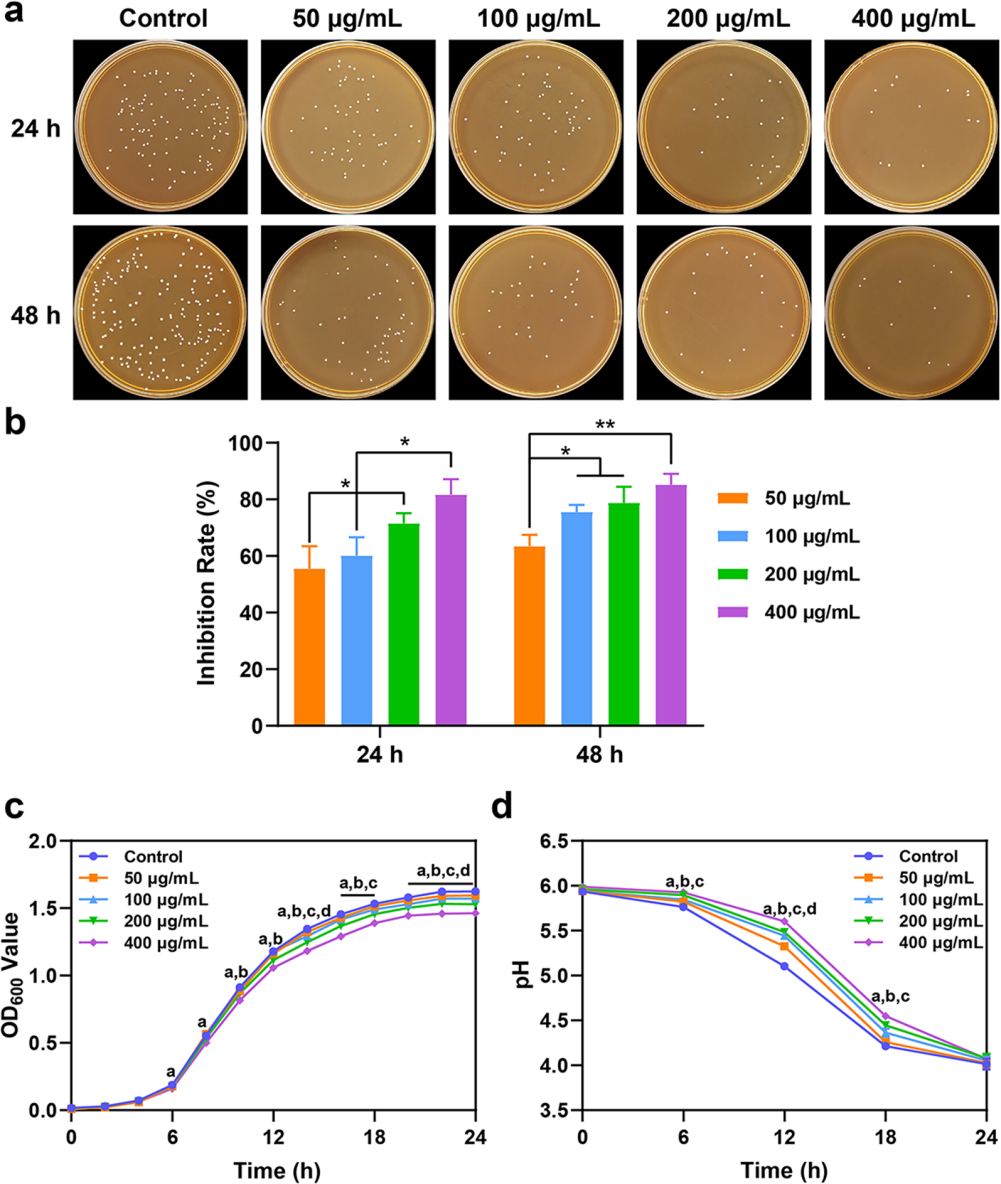
The antibacterial effect of PL-CDs incubated with *L. rhamnosus* for 24 and 48 h. **a**, **b** Photographs of *L. rhamnosus* incubated with PL-CDs at different concentrations and the quantified bacterial inhibition rate. **c**, **d** Growth curves of *L. rhamnosus* and pH changes of solution induced by PL-CDs at different concentrations during 24 h. ^a^*p* < 0.05 (400 μg/mL vs. Control); ^b^*p* < 0.05 (200 μg/mL vs. Control); ^c^*p* < 0.05 (100 μg/mL vs. Control); ^d^*p* < 0.05 (50 μg/mL vs. Control)

To observe the effect of PL-CDs on probiotic growth, PL-CDs at different concentrations were incubated with *L. rhamnosus* for 48 h. As shown in Fig. 2a, PL-CDs significantly inhibited the growth of *L. rhamnosus* in a concentration-dependent manner, in which the bacterial inhibition rates of PL-CDs at (200 or 400 μg/mL) were both higher than 70% (Fig. 2b). Moreover, the growth curve of *L. rhamnosus* was also monitored, and results showed that *L. rhamnosus* grew speedily and attained an exponential growth level after 4 h in each group. Similarly, the inhibitory effect of PL-CDs at 200 or 400 μg/mL on *L. rhamnosus* was observed obviously compared with that in the control group in the following 14 h (Fig. 2c).

Probiotics can produce SCFAs to improve energy metabolism during the metabolism process and maintain immune and intestinal homeostasis [36]. Given the reduced SCFAs levels closely correlated with type-2 diabetes, obesity, and autoimmune diseases [37, 38], the effect of PL-CDs on the metabolic capacity of *L. rhamnosus* was assessed by testing the pH change of the medium. As shown in Fig. 2d, although a slight decrease in pH values was found during the first 6 h, the pH of the solution experienced a significant decline in all groups in the following 18 h, indicating the rapid growth and metabolism of *L. rhamnosus*. Notably, the solution pH induced by PL-CDs at high concentrations (i.e., 200 and 400 μg/mL) exhibited a high level at 12 h compared with other groups.

**Fig. 3:**
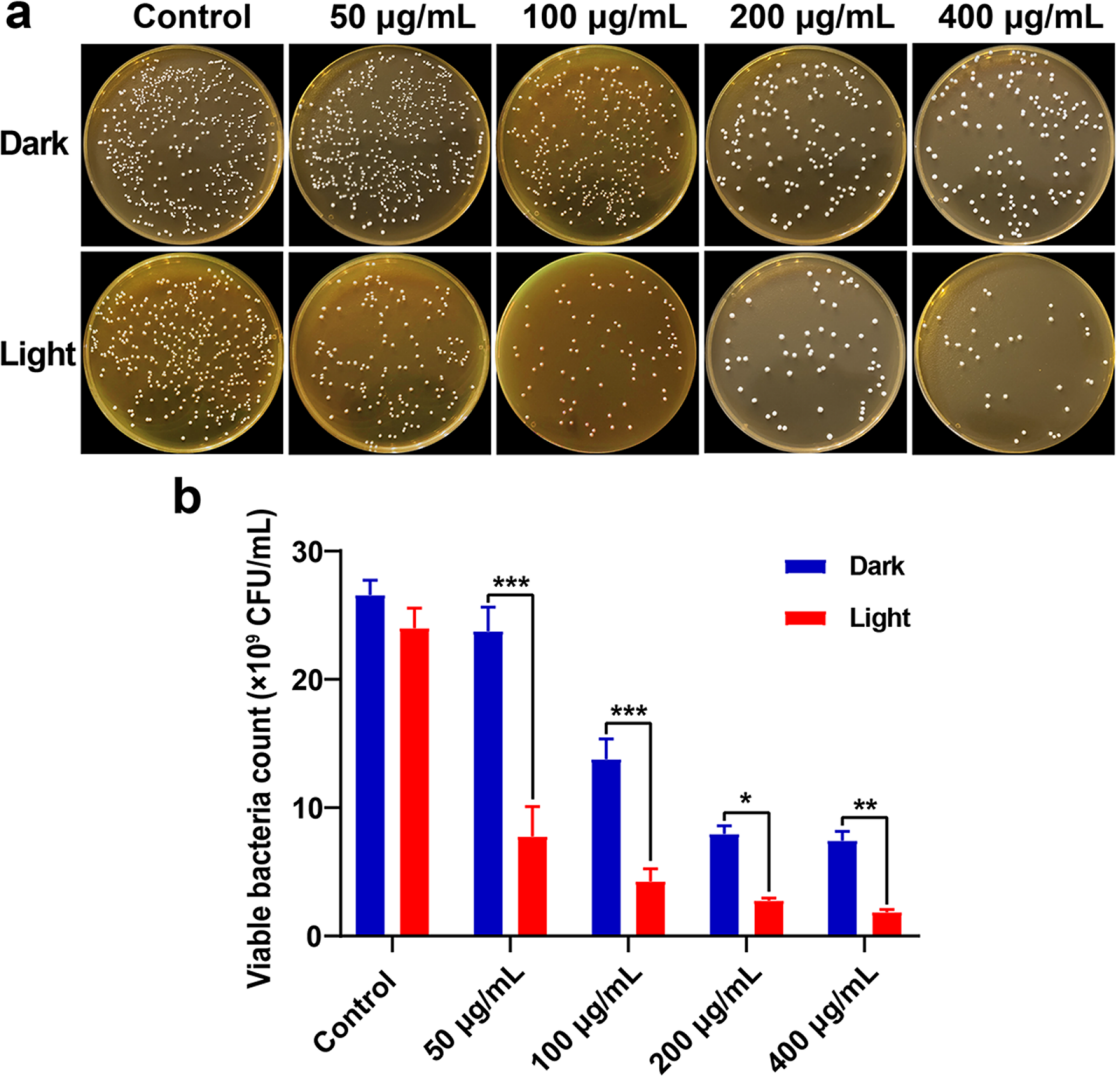
The antibacterial effect of PL-CDs incubated with *L. rhamnosus* for 24 h in the presence or absence of light. **a** Photographs of *L. rhamnosus* incubated PL-CDs at different concentrations for 24 h under dark or light conditions. **b** The quantified bacterial count from Image J

Considering the light-induced photodynamic property of CDs to kill bacteria [39], we subsequently detect whether the antibacterial activity of PL-CDs depends on light in the current study. Results showed that although the antibacterial ability is indeed more potent under light conditions, considerable antibacterial effects were still detectable even under dark conditions (Fig. 3a, b). CDs were reported to drive photoexcited state processes that can generate ROS to perform enhanced antibacterial effect [40, 41]. Thus, under light conditions, the part of the negative effect of PL-CDs on probiotics was dependent on its photodynamic property. Taken together, this study confirmed the negative effect of PL-CDs on probiotics in a concentration-dependent manner, not dependent on light.

### PL-CDs increase the elongation of probiotics

**Fig. 4:**
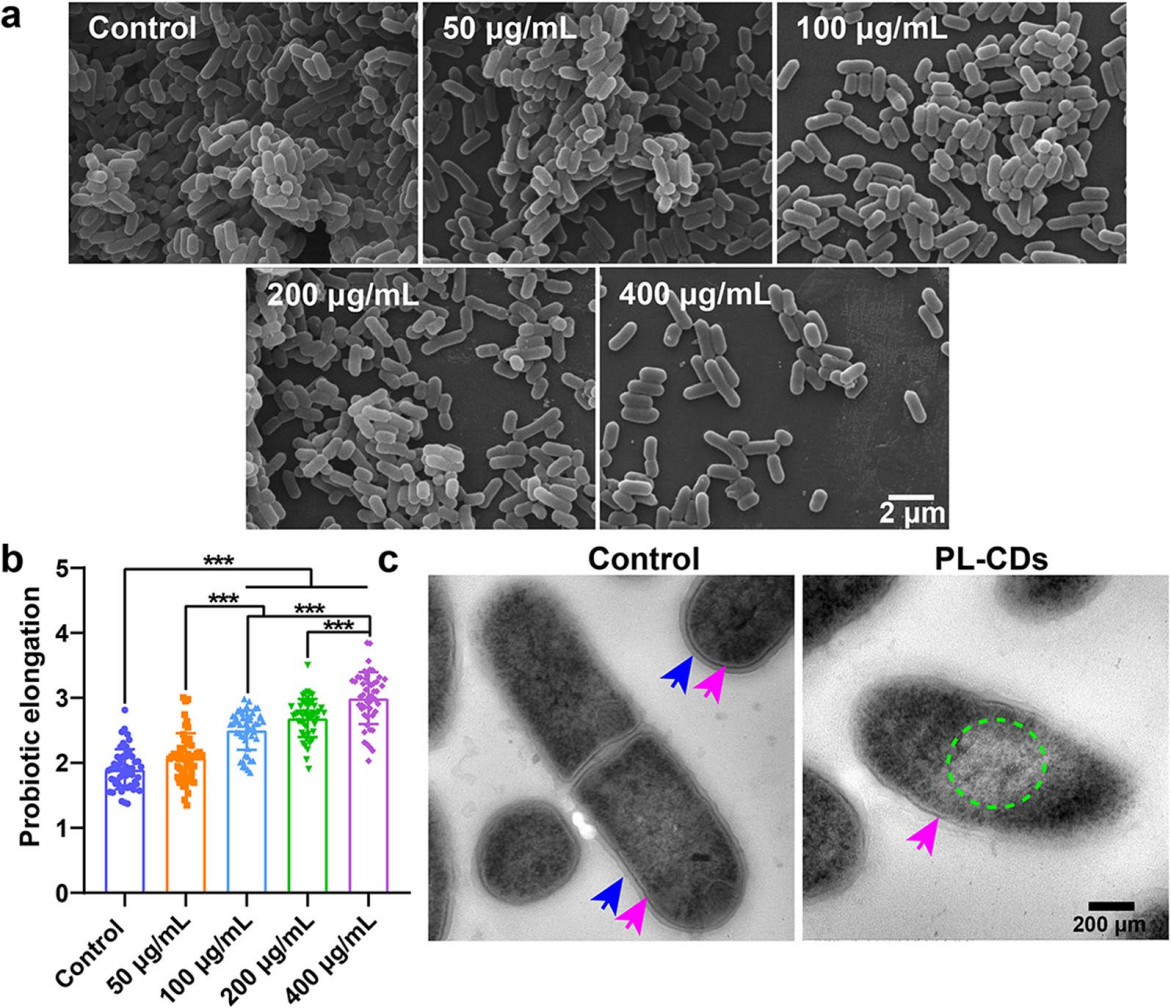
Effects of PL-CDs on morphology and internal structure of *L. rhamnosus*. **a**, **b** SEM images of *L. rhamnosus* incubated with PL-CDs at different concentrations for 24 h, and the quantified probiotic elongation (n = 50). **c** TEM images of *L. rhamnosus* incubated with PL-CDs (400 μg/mL) for 24 h. Blue and purple arrows represent the cell wall and cell membrane of *L. rhamnosus*, respectively; Green wireframe represents the cytoplasmic distribution

Since morphological changes in bacteria are common biological effects in response to variable environments involved in temperature or osmotic pressure [34], the morphology variation of *L. rhamnosus* induced by PL-CDs was detected in this study. As shown in Fig. 4a, although *L. rhamnosus* exhibited rod-like morphology in all groups, the elongation of *L. rhamnosus* was found to increase significantly with the enhanced PL-CDs concentration (Fig. 4b). In addition, the bacterial membrane of *L. rhamnosus* was disrupted with the occurrence of blurred boundaries (pointed by the arrows) and uneven cytoplasmic distribution (marked by the green wireframe) (Fig. 4c). As reported in previous studies, in response to the physical and biological stresses of the environment, bacteria were inclined to change the bacterial morphology to increase the surface area, thus enhancing the drug resistance of bacteria to avoid phagocytosis-mediated killing [42, 43]. These evidences suggest the positive role of altered bacterial morphology in facilitating cell survival. Thus, to increase the resistance of probiotics to PL-CDs, the increased elongation of *L. rhamnosus* was noted to increase the surface area in this study.

### *PL-CDs induce oxidative damage in probiotics *via* the ROS mechanism*

**Fig. 5:**
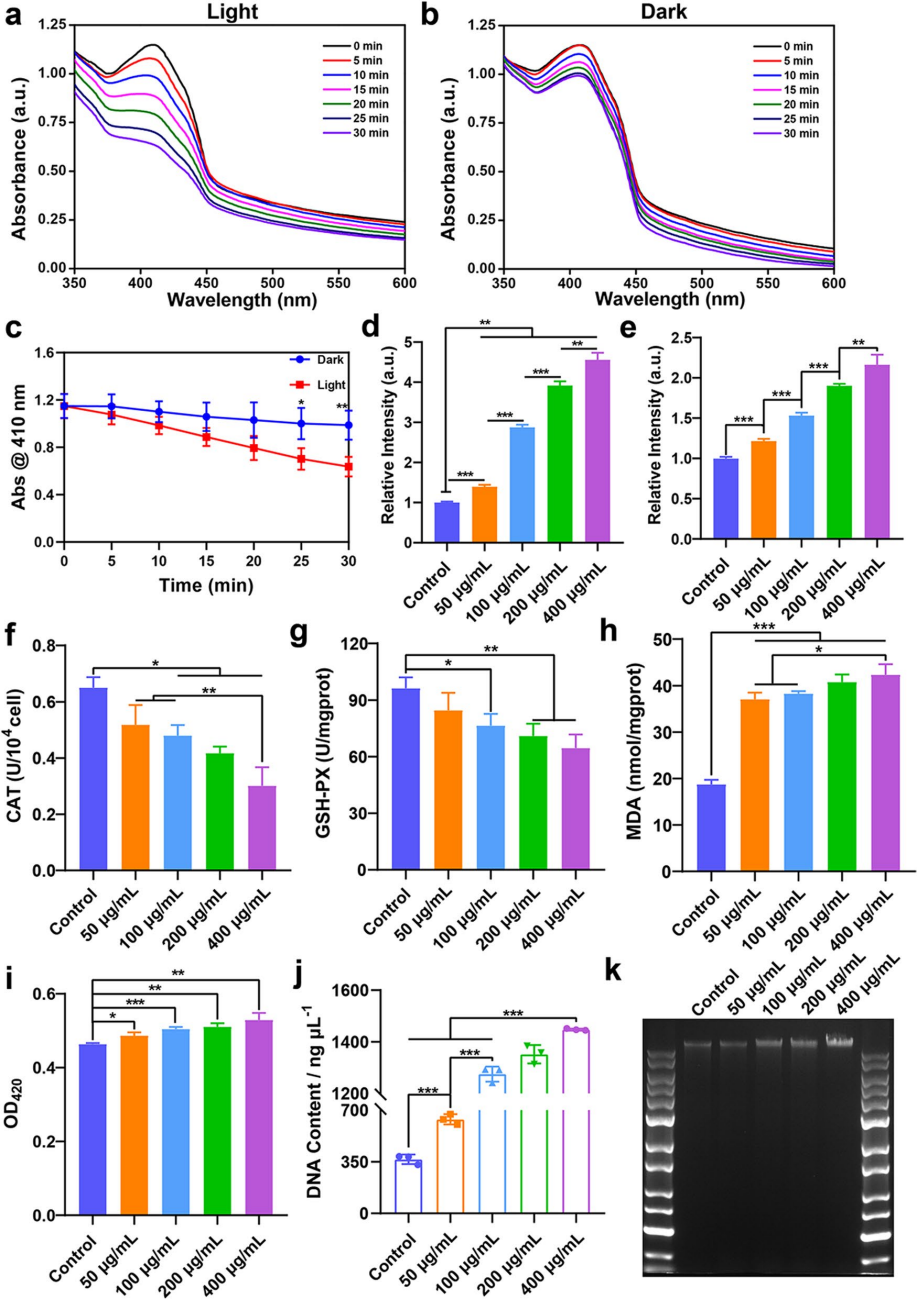
The antibacterial mechanism of PL-CDs against *L. rhamnosus.* **a**, **b** ROS generation induced by PL-CDs under light or dark conditions. **c** The absorbance of DPBF at 410 nm. **d**, **e** Intracellular ROS production of *L. rhamnosus* induced by PL-CDs under light or dark conditions. **f**–**h** The enzyme activities of CAT, GSH-PX, and MDA affected by PL-CDs. **i** Detection of membrane permeability of *L. rhamnosus*. **j**, **k** DNA content detected by NanoDrop and electrophoresis

ROS generation is one of the common mechanisms involved in the toxicity of nanomaterials [44–46], which is also associated with the antibacterial effect of most of the material. To observe the light-induced ROS production of PL-CDs, the absorbance change of PL-CDs solution containing DPBF was estimated under light or dark conditions. As shown in Fig. 5a, b, the significant decrease of absorbance peak at 410 nm indicates that light can effectively induce PL-CDs to generate ROS. Concretely, the absorbance at 410 nm decreased by 44.6% under light conditions while 14.0% under dark conditions (Fig. 5c). For the ROS generated in the probiotics, PL-CDs were verified to significantly increase the expression level of ROS in probiotics (Fig. 5d, e). Especially, ROS expression in *L. rhamnosus* incubated with 400 μg/mL PL-CDs was approximately 4.6 times higher than that in the control group under light conditions, while only 2.2 times higher under dark conditions. This evidence confirmed that the antibacterial mechanism of PL-CDs might be relevant to the excessive expression of ROS with light exposure, which results in a state of oxidative damage in bacteria.

In general, normal organisms are in balance between oxidative stress and antioxidants. Once the balance is disrupted, the activity of enzymes such as CAT and GSH-PX that eliminate ROS will change [47]. To analyze the role of the induced ROS in probiotic behavior, the enzymatic activities of CAT and GSH-PX were analyzed in this study. It was found that CAT and GSH-PX activities significantly reduced in PL-CDs-treated *L. rhamnosus* (Fig. 5f, g). Since oxidative damage could destroy the protein structure of bacteria and induce death [48], the reduced activity of CAT and GSH-PX indicated that PL-CDs could also decrease ROS elimination of probiotics to aggravate bacterial oxidative damage [49]. Given that the oxidative stress caused by ROS can induce lipid peroxidation in bacterial membranes and inhibit biofilm formation [50, 51]. MDA, a marker of lipid oxidative damage, was further detected to estimate the oxidative effect of PL-CDs-induced ROS on probiotic membranes. Results showed that the MDA level of *L. rhamnosus* was significantly enhanced by PL-CDs compared with that of the control group (Fig. 5h). Overall, these results support the conclusion that PL-CDs perform antibacterial effects on probiotics by stimulating ROS-induced oxidative damage.

Besides, bacterial membranes were selectively permeable barriers that enhance the exchange of substances inside and outside the bacteria [52]. Once the bacterial membrane is disrupted, the released β-galactosidase is inclined to hydrolyze ONPG, thereby producing a yellow chromogenic substance [53]. Therefore, ONPG was detected to evaluate the permeability of the probiotic membrane. As shown in Fig. 5i, the membrane permeability of *L. rhamnosus* was found to increase in PL-CDs groups compared with the control group. Changes in the bacterial membrane inevitably induced leakage of contents and disrupted intracellular homeostasis. The increased DNA leakage of *L. rhamnosus* was therefore found to be affected by PL-CDs in a concentration-dependent manner (Fig. 5j, k). Taken together, the antibacterial mechanism lay in the ROS and oxidative stress caused by PL-CDs, which induced lipid peroxidation and increased membrane permeability, eventually resulting in the leakage of contents.

Carbon dots are widely existing in daily life and may cause damage to the health of the organism. Zhang et al. reported that CDs from baked lamb caused cellular ROS accumulation and may induce oxidative stress in cells [54]. Boobalan et al. demonstrated that mushroom-derived CDs disrupt the integrity of bacterial membranes by generating ROS [55]. All these strongly confirmed food-borne carbon dots can produce toxic effects through ROS-induced oxidative stress. However, to the best of our knowledge, no reports were able to accurately determine the actual exposure level of the organism to CDs. Other studies had utilized certain doses of CDs to organisms, resulting in considerable oxidative stress. For example, Tang et al. reported that 1000 μg/mL CDs inhibited the growth of *Enterococcus faecalis* by damaging the bacterial wall through the production of ROS-induced oxidative stress [6]. Saravanan et al. demonstrated that CDs at 500 μg/mL induced oxidative damage to Gram-negative bacteria through the production of ROS [56]. In comparison, the current study used 400 µg/mL exposure of CDs, lower than all the above studies, which means that our study probably reflects real-world situations better. Of course, accurate determination of internal/external exposure levels of CDs would be required before a solid conclusion may be reached.

### PL-CDs inhibit cellular activity and promoted apoptosis

**Fig. 6:**
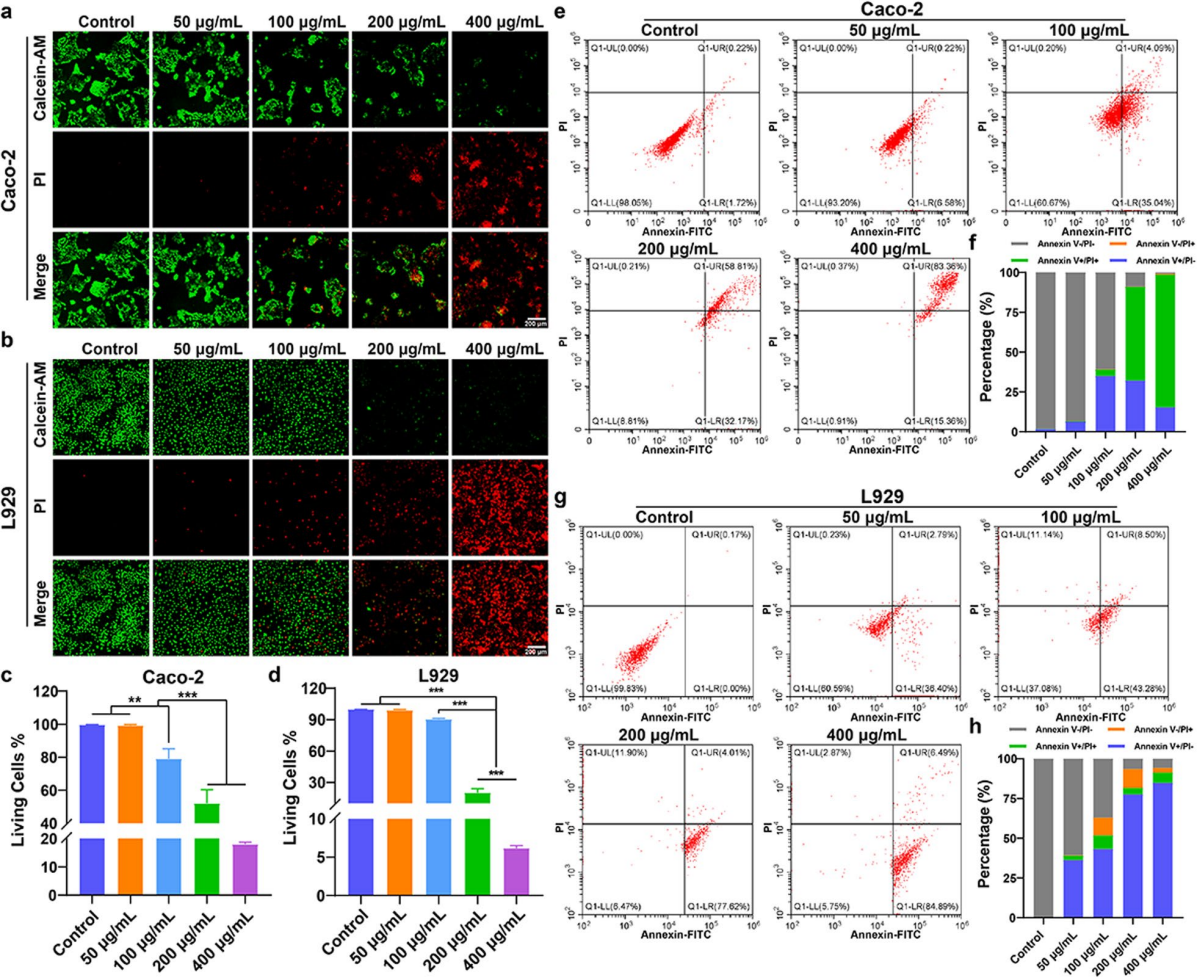
Toxic effects of PL-CDs on cells. **a**, **b** Live-dead staining of Caco-2 and L929 incubated with PL-CDs for 24 h. **c**, **d** The quantified percentage of living cells from image **J**. **e**–**h** Effect of PL-CDs on cell apoptosis of Caco-2 and L929 for 24 h, and the quantified Annexin-FITC/PI percentage

In addition to the intestinal flora, the investigation of the toxic effects of PL-CDs on normal cells is critical in the intestinal microenvironment. As shown in Fig. 6a–d, PL-CDs significantly inhibited the cell viability and growth (Additional file 1: Fig. S1) of Caco-2 and L929 in a concentration-dependent manner. To detect the effect of PL-CDs on cell apoptosis, Caco-2 and L929 cells were also incubated with PL-CDs at different concentrations. It was found that PL-CDs induced cell apoptosis in a concentration-dependent manner (Fig. 6e, g), in which the apoptosis rates of Caco-2 and L929 cells treated by 400 μg/mL PL-CDs reached 98.72% and 91.38%, respectively (Fig. 6f, h). Overall, these results suggest the potential toxicity of PL-CDs for inhibiting cellular activity and promoting cell apoptosis.

### *PL-CDs induce intestinal inflammation *in vivo

**Fig. 7:**
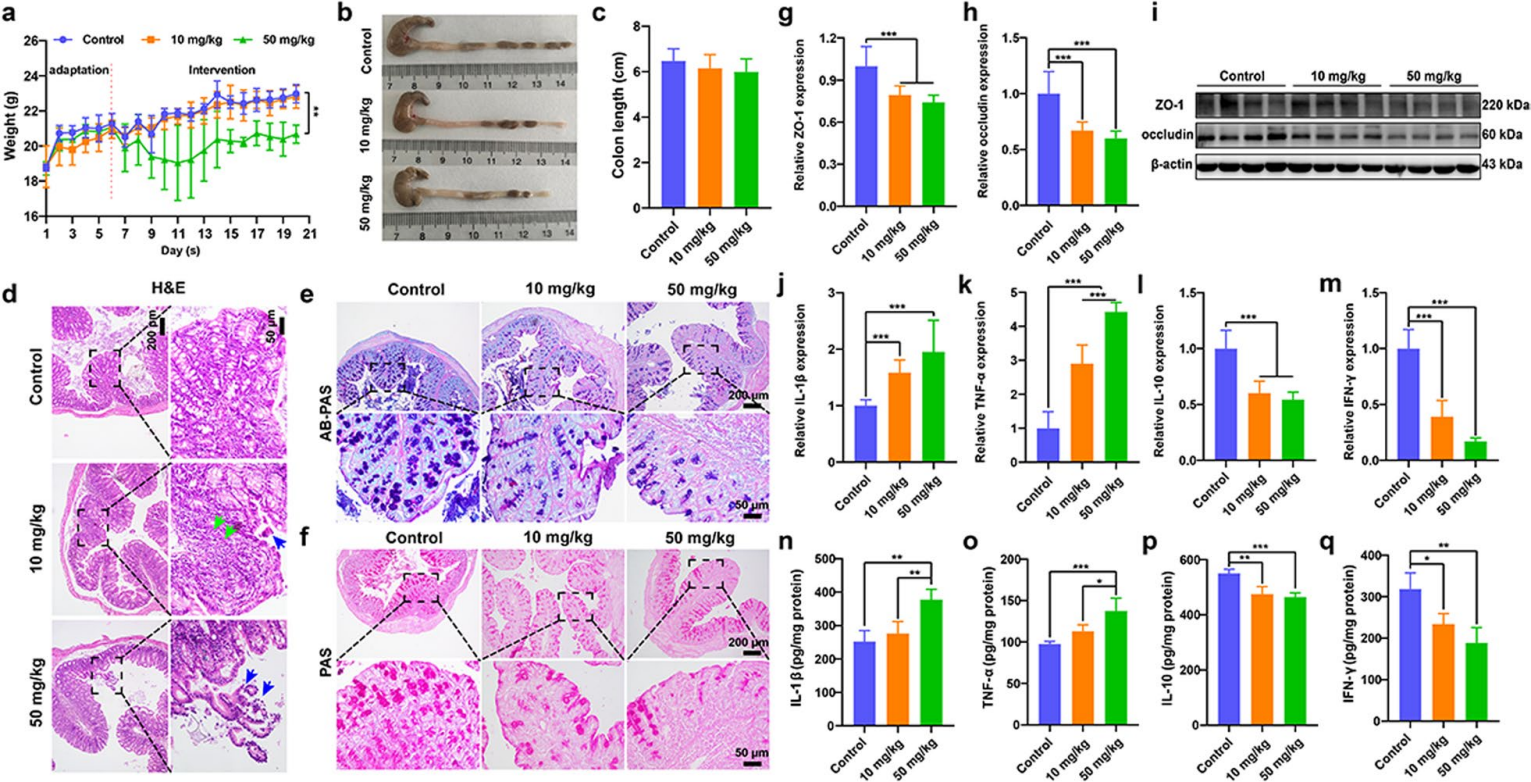
Intestinal pathological damage and inflammation caused by PL-CDs. **a** Monitoring of mouse weight affected by PL-CDs. **b**, **c** Images of the colon and the quantified colon length. **d**–**f** Images of H&E, AB-PAS, and PAS staining of colon tissue. **g**–**i** mRNA and protein expressions of tight junction proteins (ZO-1 and occludin) by qRT-PCR and Western blot. **j**–**q** The mRNA and protein expressions of pro-inflammatory (IL-1β and TNF-α) and anti-inflammatory factors (IL-10 and IFN-γ) by qRT-PCR and ELISA

To detect the intestinal toxicity induced by PL-CDs in vivo, mice were gavaged with saline, 10 mg/kg or 50 mg/kg PL-CDs. Although the weight of mice exhibited no significant difference in the 10 mg/kg group compared to the control group, the decreased weight was found in the 50 mg/kg group during 21 days of raising time (Fig. 7a). Meanwhile, the high content of PL-CDs tended to decrease the average colon length of mice (Fig. 7b, c). Nevertheless, the organ coefficient results showed no significant changes in the heart, liver, spleen, lung, kidney, brain, thymus, and colon (Additional file 1: Fig. S2a–h). This indicates that PL-CDs are inclined to only affect intestine remodeling. To further observe the pathological changes of the colon, PL-CDs were verified to not only result in intestinal epithelial damage, villi shedding, and inflammatory infiltration (Fig. 7d), but also significantly reduce the number of goblet cells and glycoprotein in the colon tissue (Fig. 7e, f). However, no obvious toxic effects were found on the heart, liver, spleen, lung, and kidney (Additional file 1: Fig. S2i). Given the critical role of the intestinal barrier in regulating intestinal inflammation [57, 58], the expressions of barrier-related factors (ZO-1 and occludin) were further examined. Similarly, both ZO-1 and occludin expression significantly decreased after the treatment of PL-CDs, indicating the damage of the intestinal barrier (Fig. 7g–i). To verify the intestinal inflammation, the expression levels of inflammatory factors were also examined and results showed that PL-CDs significantly up-regulated the expressions of IL-1β and TNF-α while down-regulated the expressions of IL-10 and IFN-γ, thus confirming the presence of intestinal inflammation induced by PL-CDs (Fig. 7j–q).

Inflammation directly damages intestinal health. The persistent development of intestinal inflammation can induce water-electrolyte disturbance, intestinal ecological dysregulation, intestinal barrier dysfunction, and bacterial translocation [59]. Thus, the above results suggest that PL-CDs may induce intestinal toxicity by producing pathological damage to the intestine, disrupting the intestinal barrier, and causing intestinal inflammation.

### PL-CDs result in intestinal flora imbalance in vivo

**Fig. 8:**
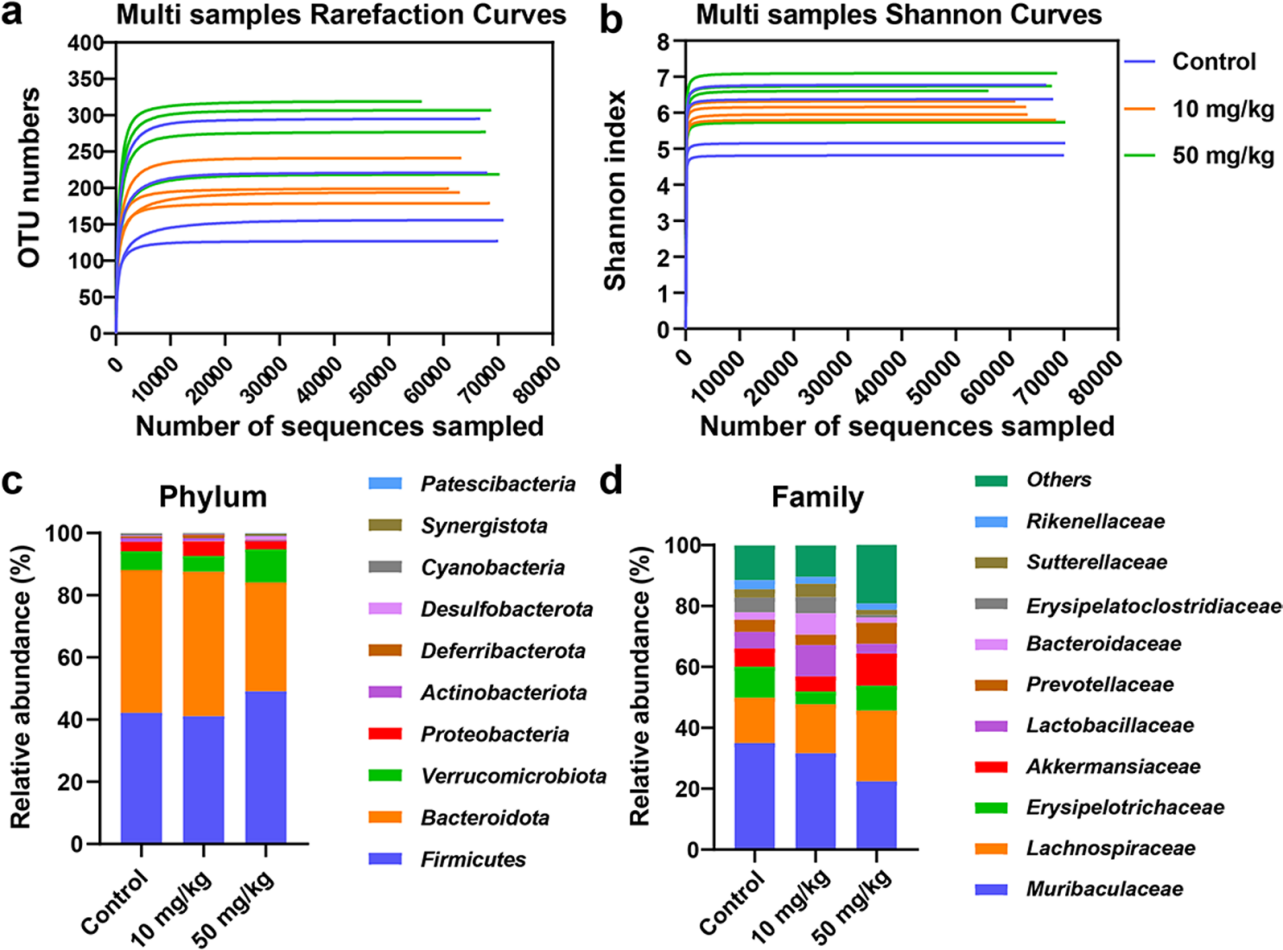
Intestinal flora imbalance induced by PL-CDs. **a**, **b** Multi samples rarefaction curves and Shannon curves. **c**, **d** Relative abundance of intestinal flora at the phylum level and at the family level, n = 4

IBD is closely associated with dysbiosis of the intestinal flora [60]. To analyze the role of inflammation caused by PL-CDs in destroying the balance of intestinal flora, 16S rRNA gene sequencing was performed in this study. The flat trends of all the sample dilution curves indicate the suitable amount of sequencing data to reflect species diversity (Fig. 8a). The enhanced Shannon index indicates the increased species abundance in PL-CDs groups (Fig. 8b). Besides, at the phylum level, *Bacteroidota* and *Firmicutes* were the main phylum in the intestinal flora. However, the relative abundance of *Firmicutes* increased while those of *Bacteroidota* decreased after the treatment of PL-CDs (Fig. 8c). Moreover, PL-CDs significantly reduced the relative abundance of *Muribaculaceae* while increasing those of *Lachnospiraceae* compared with the control group (Fig. 8d).

In the intestinal microenvironment, probiotics and harmful bacteria maintain the balance of the intestinal flora [61], in which probiotics are involved in carbohydrate metabolism to provide energy to intestinal epithelial cells [62], and harmful bacteria produce lipopolysaccharides or endotoxins to induce inflammation [63]. Previous studies have proved that allogenic material can induce intestinal toxicity by breaking intestinal flora balance [64], and the associated main phylum of the intestinal flora is *Firmicutes*, *Bacteroidota*, *Proteobacteria*, and *Actinobacteria* [65]. Among them, the ratio variation of *Firmicutes* to *Bacteroidota* (F/B) represented flora imbalance [66]. For example, the increased F/B ratio, which is correlated with a chronic inflammatory state, occurred in the aged mice on a high-fat diet [67]. Hence, the increased ratio of F/B in this study indicates that PL-CDs induce intestinal inflammation. Furthermore, *Muribaculaceae*, as the main intestinal flora in healthy individuals, has been reported to produce acetate and propionate to facilitate the regulation of intestinal pH and maintain intestinal homeostasis [68]. Additionally, the relative abundance of *Muribaculaceae*, including *Lactobacillus*, was verified to reduce in the colitis model [69], and *Lachnospiraceae*, as harmful bacteria, inhibit the production of butyrate [70]. In this study, the reduced relative abundance of *Muribaculaceae* and increased that of *Lachnospiraceae* in PL-CDs groups strongly support the conclusion that PL-CDs mediated the imbalance of intestinal flora to induce intestinal inflammation.

**Fig. 9:**
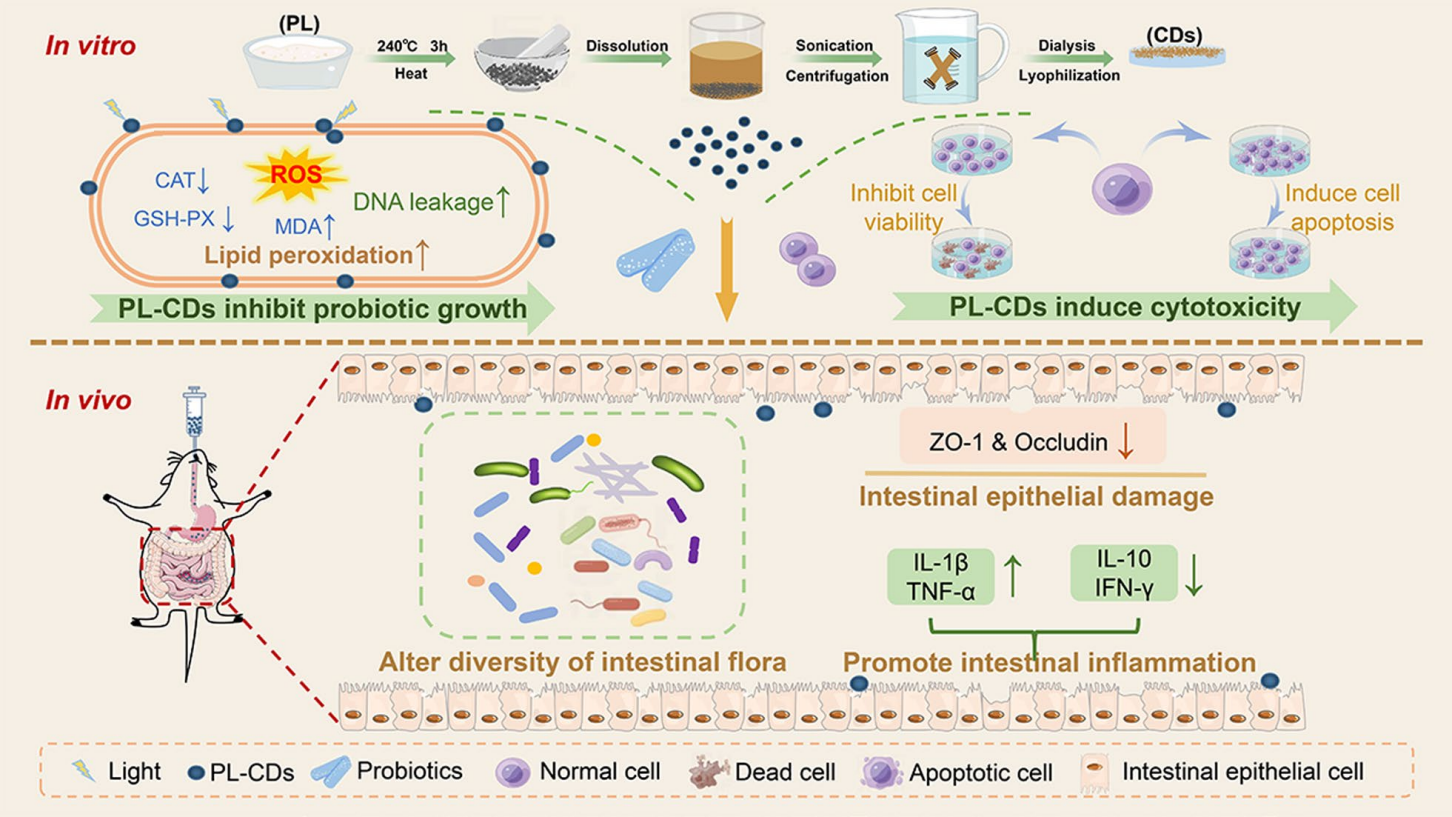
Model of PL-CDs induced intestinal toxicity

In summary, PL-CDs inhibited probiotic growth by stimulating ROS-induced oxidative stress and decreasing the antioxidant level of probiotics, thus damaging the bacterial membrane via generating lipid peroxidation. In vivo, the excessive intake of PL-CDs not only exhibits the risk of inhibiting cellular activity and promoting apoptosis, but also may contribute to the intestinal flora imbalance by altering intestinal flora diversity, damaging the intestinal barrier, and inducing intestinal inflammation (Fig. 9). Overall, this study provides effective and insightful evidence for the side effects of PL-CDs on the intestinal microenvironment, implying the necessity to reduce the exposure of CDs to the intestine. Nevertheless, the signaling pathway of CDs-induced intestinal damage needs to be further investigated, which will facilitate the comprehensive prevention and treatment of intestinal diseases.

## Conclusions

In the study, PL-CDs with -NH_2_ were successfully prepared by pyrolysis, and it was first demonstrated that the positively charged PL-CDs have negative effects on probiotics. The antibacterial mechanism was that PL-CDs increased the production of ROS and decreased the activity of antioxidant enzymes in probiotics, which resulted in the production of MDA, the increase of membrane permeability, and the release of DNA, eventually leading to the death of probiotics. Moreover, PL-CDs inhibited the activity of intestinal epithelial cells and induced cell apoptosis in vitro, meanwhile causing intestinal flora dysbiosis and intestinal inflammation in vivo, ultimately leading to pathological damage to the intestine.

## Supplementary Information

**Additional file 1. **Supplementary material and method: Cell activity assay and **Fig S1**. Effect of PL-CDs on cell activity. a Relative viability of PL-CD incubated with Caco-2 for 1, 3, and 5 days. b Relative viability of PL-CD incubated with L929 for 1, 3, and 5 days. **Fig S2**. Effects of PL-CDs on other organs. a-h Organ coefficients of the heart, liver, spleen, lung, kidney, brain, thymic, and colon. i H&E images of heart, liver, spleen, lung, and kidney.

**Additional file 2.** The primer sequences used in this study.

## Data Availability

The data that support the findings of this study are available from the corresponding authors upon reasonable request.

## Abbreviations

CDs: Carbon dots
PL: ε-Poly-l-lysine
*L. rhamnosus*: *Lactobacillus rhamnosus*
ROS: Reactive oxygen species
F/B: Firmicutes to Bacteroidota
*E. coli*: *Escherichia coli*
*S. aureus*: *Staphylococcus aureus*
SCFAs: Short-chain fatty acids
IBD: Inflammatory bowel disease
TEM: Transmission electron microscope
UV–vis: Ultraviolet–visible
FT-IR: Fourier transform infrared
MRS: Man–Rogosa–Sharpe
SEM: Scanning electron microscope
DPBF: 1,3-Diphenylisobenzofuran
DCFH-DA: 2′,7′-Dichlorodihydrofluorescein diacetate
CAT: Catalase
GSH-PX: Glutathione peroxidase
MDA: Malondialdehyde
ONPG: *O*-Nitrophenyl β-galactoside
H&E: Hematoxylin and eosin
PAS: Periodic acid-schiff
AB-PAS: Alcian blue-periodic acid-shiff
RIPA: Radioimmunoprecipitation
SDS-PAGE: Sodium dodecyl sulfate polyacrylamide gel electrophoresis
PVDF: Polyvinylidene fluoride
ZO-1: Zonula occludens-1
HRP: Horseradish peroxidase
ELISA: Enzyme-linked immunosorbent assay
IL: Interleukin
TNF: Tumor necrosis factor
IFN: Interferon
QIIME2: Quantitative insights into microbial ecology 2
OTUs: Operational taxonomic units
