# Supplementary material for: Carbon dots induce pathological damage to the intestine via causing intestinal flora dysbiosis and intestinal inflammation
Source: J Nanobiotechnology. 2023 May 25;21:167. doi: 10.1186/s12951-023-01931-1 (PMC10210306; doi:10.1186/s12951-023-01931-1)
Supplement: Supplementary file 1 — Additional file 1. Supplementary material and method: Cell activity assay and Fig S1. Effect of PL-CDs on cell activity. a Relative viability of PL-CD incubated with Caco-2 for 1, 3, and 5 days. b Relative viability of PL-CD incubated with L929 for 1, 3, and 5 days. Fig S2. Effects of PL-CDs on other organs. a-h Organ coefficients of the heart, liver, spleen, lung, kidney, brain, thymic, and colon. i H&E images of heart, liver, spleen, lung, and kidney. [file 12951_2023_1931_MOESM1_ESM.docx]

**Additional file 1**

**Carbon dots induce pathological damage to the intestine *via* causing intestinal flora dysbiosis and intestinal inflammation**

Mengmeng Jia ^a, 1^, Bingcheng Yi ^b, 1^, Xian Chen ^a^, Yongzhi Xu ^c^, Xinkai Xu ^c^, Zhaoxu Wu ^a^, Jing Ji ^a^, Dianke Yu ^a^, Yuxin Zheng ^a^, Qihui Zhou *^b,c,d^, Yanjie Zhao *^a^

**Author Affiliations:**

^a^ School of Public Health, Qingdao University, Qingdao 266071, China.

^b^ School of Rehabilitation Sciences and Engineering, University of Health and Rehabilitation Sciences, Qingdao 266071, China.

^c^ School of Stomatology, Qingdao University, Qingdao 266003, China.

^d^ Zhejiang Engineering Research Center for Tissue Repair Materials, Wenzhou Institute, University of Chinese Academy of Sciences, Wenzhou, Zhejiang 325000, China.

*Corresponding author:

Qihui Zhou

Email: qihuizhou@uor.edu.cn

Yanjie Zhao

Email: zhaoyj@qdu.edu.cn

^1^ Mengmeng Jia and Bingcheng Yi contributed equally to this work.

**Materials and methods**

**Cell activity assay**

Cell viability was assessed by cell counting kit-8 (CCK-8) (Dojindo, Japan). Briefly, Caco-2 and L929 cells were seeded in 96-well plates at a density of 3000 cells/well. After 24 h, different concentrations of PL-CDs were added to wells and incubation with cells for 1, 3, and 5 d. Then 10% CCK-8 solution was added and incubated at 37℃ for 2 h. The absorbance of OD_450_ was detected by a microplate reader (Thermo Fisher Scientific, Beijing, China). This experiment was set up with four replicate wells and repeated three times.


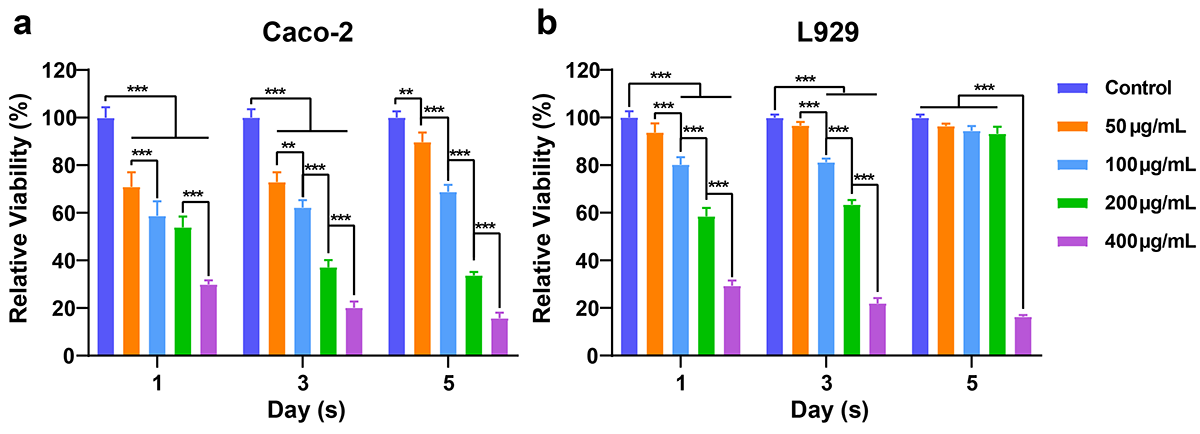


**Fig S1. Effect of PL-CDs on cell activity. a** Relative viability of PL-CD incubated with Caco-2 for 1, 3, and 5 days. **b** Relative viability of PL-CD incubated with L929 for 1, 3, and 5 days.

**
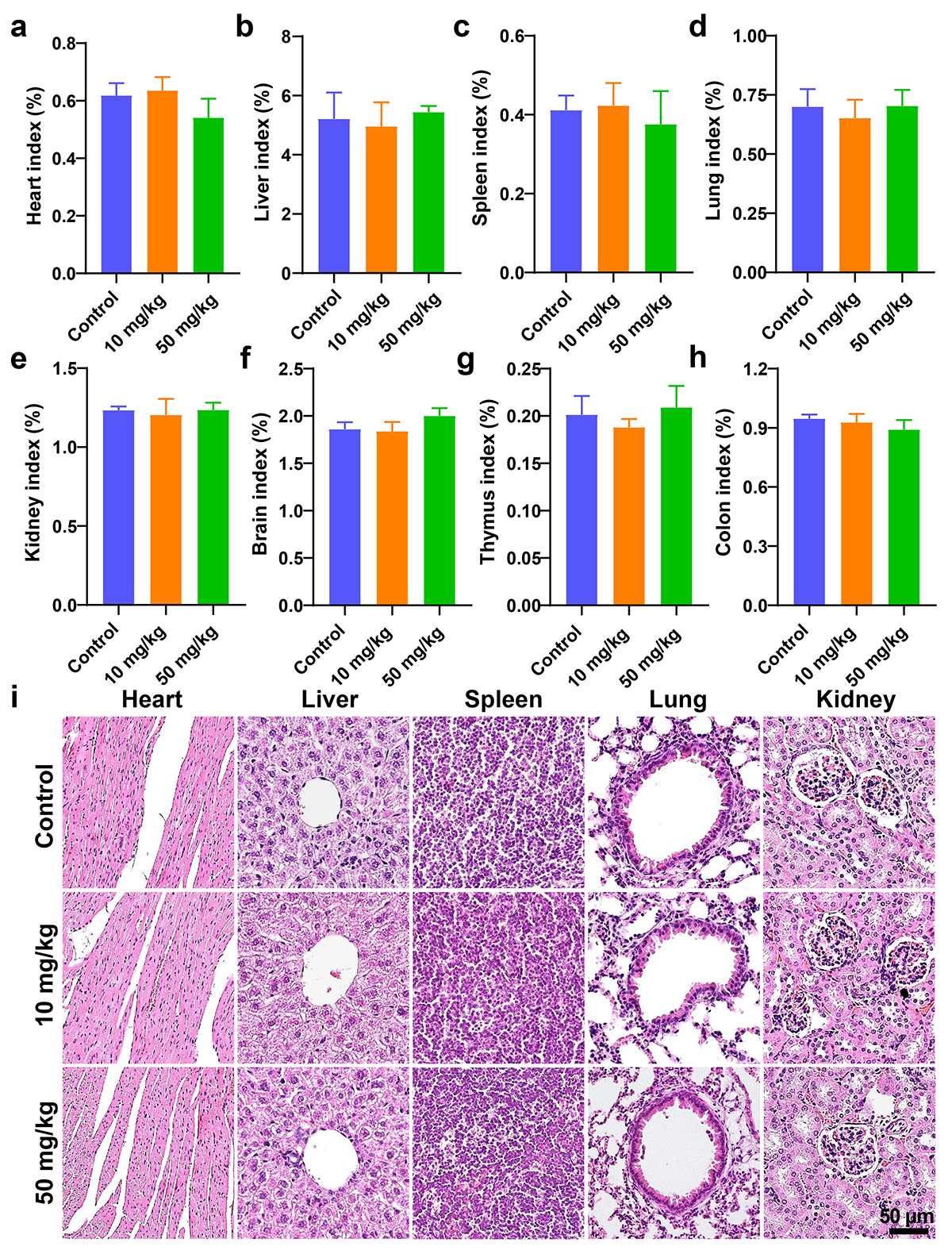
**

**Fig S2.** **Effects of PL-CDs on other organs.** **a-h** Organ coefficients of the heart, liver, spleen, lung, kidney, brain, thymic, and colon. **i** H&E images of heart, liver, spleen, lung, and kidney.
